# Study on Sensing Urine Concentrations in Water Using a Microwave Sensor Based on Hilbert Structure

**DOI:** 10.3390/s24113528

**Published:** 2024-05-30

**Authors:** Rusul Khalid Abdulsattar, Musab T. S. Al-Kaltakchi, Iulia Andreea Mocanu, Amer Abbood Al-Behadili, Zaid A. Abdu Hassain

**Affiliations:** 1 Department of Electrical Engineering, University of Technology, Baghdad 10066, Iraq; rusul.k.abdulsattar@uotechnology.edu.iq; 2 Department of Electrical Engineering, College of Engineering, Mustansiriyah University, Baghdad 10052, Iraq; m.t.s.al_kaltakchi@uomustansiriyah.edu.iq (M.T.S.A.-K.);; 3 Telecommunications Department, National University of Science and Technology Politehnica Bucharest, 060042 Bucharest, Romania

**Keywords:** microwave sensor, fractal structure, water content, urine

## Abstract

In this study, a two-port network-based microwave sensor for liquid characterization is presented. The suggested sensor is built as a miniature microwave resonator using the third iteration of Hilbert’s fractal architecture. The suggested structure is used with the T-resonator to raise the sensor quality factor. The suggested sensor is printed on a FR4 substrate and has a footprint of $40\times 60\times 1.6\;{\mathrm{mm}}^{3}$. Analytically, a theoretical investigation is made to clarify how the suggested sensor might function. The suggested sensor is created and put to the test in an experiment. Later, two pans to contain the urine Sample Under Test (SUT) are printed on the sensor. Before loading the SUT, it is discovered that the suggested structure’s frequency resonance is 0.46 GHz. An 18 MHz frequency shift is added to the initial resonance after the pans are printed. They monitor the S-parameters in terms of S12 regarding the change in water content in the urine samples, allowing for the sensing component to be completed. As a result, 10 different samples with varying urine percentages are added to the suggested sensor to evaluate its ability to detect the presence of urine. Finally, it is discovered that the suggested process’ measurements and corresponding simulated outcomes agreed quite well.

## 1. Introduction

The motivation for sensing the urine concentrations in water is due to the effects of urine concentration on four different conditions. The four factors leading to concentrated urine involve various health conditions, including heart failure, fluid loss through diarrhea or excessive sweating, narrowing of the kidney artery, presence of sugar or glucose in the urine, and Syndrome of Inappropriate Antidiuretic Hormone Secretion (SIADH). In [1], a brand-new metamaterial structure is created for applications requiring sensing in the 8–12 GHz region. With the use of this design, it was feasible to vary the transmission coefficient (S21) and reflection coefficient (S11), which were then used to distinguish between branded and unbranded diesel. The transmission coefficient (S21) and resonance frequency of this sensor exhibit notable variations that make it easy to identify. This innovative design is affordable, extremely sensitive, and readily fabricate for application in a wide frequency range from 1 to 20 GHz.

In the study in [2], a novel resonator aimed at characterizing blood glucose levels in diabetic individuals utilizes a unique setup comprising four Minkowski Open Loops (MOLs) unit cells connected to an Open-Stub Transmission Line (OSTL) featuring a Carbon Nanotubes Patch (CNT) attachment. This innovative structure is fabricated on an FR4 substrate to achieve resonance at a frequency of 2.45 GHz. To elucidate the functionality of the proposed sensor, a circuit model is developed, followed by rigorous validation using two numerical software programs, CST (v2024) and High-Frequency Simulation Software (HFSS) (v2024 R1). Subsequently, a sensitivity analysis is conducted using 15 blood samples upon completion of the sensor design, marking a significant step forward in diabetic care technology. A K-Nearest Neighbor (KNN) method is used in the sensing process to quantify the glucose level.

Researchers later improved the approach of water detection in crude oil by extending microwave technologies [3,4]. In [5], there are details on the creation and testing of a microwave and gamma-ray MFM on the oil production facilities on WAPET’s Thevenard Island from September to December 1993, as well as the creation of a microwave MFM for testing on ESSO’s West Kingfish platform in 1994. A sensor for measuring specific admittance to determine the water content of an oil-in-water emulsion is shown in [6]. The ability to quantify the water content of crude oil using an ultra-short wave technique based on a parallel line sensor has been established in [7]. Its key feature, the fusion measurement of permittivity and electrical conductivity, along with the straightforward sensor design and wide measuring range, makes field installation simple. The technique adjusts to the conditions in the oil field. Copper nanorods were used in [8] to create a unique wireless gas sensor that operates in the microwave frequency range. To discriminate between different gases at the 362 Elwi and Khudhayer air pressure, the suggested sensor is made up of copper nanorod arrays produced using the GLAD process on a traditional Microstrip antenna. The sensor’s performance was evaluated and contrasted with an antenna sample devoid of copper nanorods.

In the work in [9], a capacitance is exploited to a phase conversion circuit for the measurement of crude oil with extremely low water content. To get around the aforementioned restrictions and provide more reliable output measurements, it also offers a differential sensing approach. Then, a straightforward phase readout system was suggested to do away with the requirement for an Analog-to-Digital Converter (ADC). The article [10] illustrates a unique application for wireless passive sensor applications employing a metamaterial (MTM)-based monopole antenna construction operating at the microwave frequency band. The interdigital capacitor (Cint) and route incorporated into a traditional monopole antenna construction, which decreased the antenna size to 45% at 0.65 GHz, make up the majority of the sensor.

The study in [11] uses a non-destructive method based on a microstrip sensor to quantify the electromagnetic characteristics of gasoline products and assess the potential level of the moisture content ratio. As a result, the moisture content is the most important factor in determining the quality of various petroleum products, including mineral oil, heavy oil, lubricant, and crude oil. In the paper [12], a sensor based on a resonator-based metamaterial structure is built, simulated, prototyped, and analyzed. A ground frame surrounds the resonator, which is activated by a 50 × Feeding Transmission Line (FTL) on the substrate’s reverse side. Instead of employing waveguides to test the metamaterial, standard laboratory equipment is used. The metamaterial exhibits negative permittivity and negative permeability in the operational region, and the findings from modeling and experimentation correspond pretty well.

In [13], a microwave sensor for the nondestructive assessment of dielectric substrates is designed using a Complementary Circular Spiral Resonator (CCSR). The suggested sensor’s magnetic permeability, effective electric permittivity, and electric field concentration are all numerically determined. A sensor is constructed, and a vector network analyzer (AV3672 series) is used to measure the transmission coefficient’s (S21) magnitude. The manufactured sensor is covered with five dielectric materials (the Materials Under Test, or MUTs), and as a result of their interaction, the S21 of the sensor is measured. A loaded line CRLH TL phase shifter employing ferroelectric varactors as an adjustable load element is shown for the first time in the article [14].

The Composite Right-Left-Handed (CRLH) TL phase shifter is made to have flat frequency dependency around the center frequency when bias is applied to the differential phase shift. The idea has been theoretically and empirically proved. A brand-new methodology for complete electromagnetic characterization of magneto-dielectric materials was put out in [15] and is based on a brand-new Complementary Split-Ring Resonator CSRR sensor. The suggested sensor benefits from easy sample preparation and straightforward, low-cost production utilizing Printed Circuit Board (PCB) manufacturing technology.

In addition, the Complementary Circular Spiral Resonator (CCSR), which runs at about 2.4 GHz, is used in the study in [16] to describe a low-cost, compact, and highly sensitive microwave sensor for recognizing liquid samples and calculating their dielectric constants. By detecting the resonant frequency of the CCSR, the suggested sensor was constructed and tested to accurately identify various liquids often used in everyday life and to quantify the amounts of various ethanol–water mixes. Moreover, the liquid samples are contained within regular glass capillary tubes, and the suggested CSRR sensor operates [17] at a frequency of around 2.4 GHz. It is possible to rapidly and easily switch the capillary tubes as they travel through the inside of the CSRR structure and transmission line (in the typical direction). Consequently, it is a good option for field measurements. Furthermore, to create a small, submersible sensor, a new, open-ended microstrip structure based on the CSRR is suggested in the letter [18].

The suggested sensor differs from previously reported conventional CSRR-loaded microstrip-based sensors in that the CSRR is directly etched on the top side of the open-terminated flared microstrip line. A sensor for measuring the complex dielectric constant of materials was introduced in [19] by adjusting both the depth and frequency of a resonator-loaded line, enabling a unique method to calculate the complex dielectric constant. Unlike conventional approaches, this methodology relies on an analytical method, eliminating the need for calibration. While the concept of utilizing notch depth and frequency change for sensing is not new, it offers a fresh perspective. Ref. [20] introduced a rectangular CSRR device designed, fabricated, and tested as a sensor for detecting the thickness and dielectric constant of multilayer materials. The proposed method leverages the shift in the least transmission frequency, S21, caused by variations in sample material thickness and permittivity. The quasi-static nature of CSRR, combined with the precision of resonator-based material characterization methods, yields reliable sensors that are simple, fast, and cost-effective to manufacture. Moreover, CSRR sensing is straightforward, accurate, and computationally manageable.

A circuit model for microstrip lines loaded with electrically connected CSRR pairs was suggested and validated in [21]. An in-depth analysis of the model, which applies to both symmetric and asymmetric structures, has been conducted. The similarities to CPW transmission lines loaded with pairs of magnetically linked SRRs have also been noted. The design, construction, and testing of two antennas in various configurations with and without SRR loading may be found in [22]. Split Ring Resonators (SRRs) loading on the traditional unloaded monopole antennas produced a wider bandwidth and improved radiation characteristics. A lower resonance frequency mode that corresponds to the resonance frequency of the monopoles is excited by the SRR-loaded antennas.

In the work in [23], a tiny traveling-wave antenna is created on a single layer using a CRLH-MTM transmission line. Interdigital capacitors and dual-spiral inductive slots, where the slot is incorporated into the transmission line, are used in the construction of the CRLH MTM transmission line (TL). The CRLH MTM-TL equivalent lumped element model is initially examined. The findings from the simulation and the measurement exhibit strong agreement with one another.

An affordable and simple-to-fabricate device is suggested in the study in [24] to precisely describe liquid samples based on their dielectric characteristics. This study demonstrates the operation of an affordable, label-free microwave sensor based on MCSRR (Multiple Complementary Split Ring Resonator) technology, operating within the 2.45 GHz frequency range of the Industrial Scientific and Medical (ISM) band. Through meticulous experimentation aided by a precise numerical model, the sensor’s ability to investigate the dielectric properties of a binary mixture of water and ethanol is examined. Additionally, various samples of pure liquids with differing and higher dielectric constants compared to the binary mixture are employed to further validate the sensor’s sensitivity towards changes in the dielectric properties of the liquid being analyzed.

This paper describes a rectangular fractal geometry for characterizing urine that is based on a microwave resonator. The fractal T-shaped SRR is put onto a 50 Ω transmission line to create the resonator. It has been discovered that the suggested sensor is quite helpful for determining the percentage of urine content in water. The suggested microwave sensor circuitry’s resonance frequency of 0.46 GHz is produced by the fractal architecture. To improve selectivity, fractal geometry is employed to enlarge the effective interaction area with the Sample Under Test (SUT), creating a significant region for the electric field fringing. This enhancement shows remarkable alignment with experimental results. Evaluating the sensor’s performance entails conducting experiments where different quantities of water are added to urine. The measurements show that the urine percentages in the water are correctly identified with an overall error of less than 6% (where the overall error is calculated by the difference between the estimated value when the urine percentage is 100% in terms of resonant frequency and the actual value when the urine percentage is 0% to the actual value. According to this form: Overall error = (estimated value − actual value)/(actual value)). To be directly exposed to the electric field fringes emanating from the fractal geometry, the considered SUT is filled in the FR4 cartage pan that is positioned on the sensor surface. To measure the transmission spectra (S21) with the introduction of various SUTs, a Vector Network Analyzer (VNA) is attached to the sensor. Finally, experimental validation of the theoretical conclusions of the suggested sensor performance demonstrates great agreement with their relatives.

Here are the main contributions of this paper, summarized as follows:

- We’ve tapped into the special features of the Hilbert cell to forecast the percentage of urine drops in water across a hundred samples, ranging from one percent to a hundred percent. The unique blend of qualities in Hilbert cells—like compactness, multiband capability, minimal signal loss, consistent phase behavior, ease of integration, reduced interference, and adaptable design—makes them an appealing option for various microwave engineering tasks [25].
- A key highlight of our study is the simplicity of constructing the pan without the need for a water pump or intricate piping within the substrate.
- Our proposed system outperforms all comparable works in terms of resonator type, resonant frequency, SUT, area, and s-parameters.

The structure of this article is as follows: Section 1 presents the introduction, Section 2 shows the related work, and Section 3 includes the main structure of the proposed work of this paper. Section 5 describes the geometric details of the sensor, followed by the presentation of the numerical analysis in Section 6, the experimental results and discussion in Section 7, and finally the conclusions in Section 8.

## 2. Related Work

In the work in [26], researchers created a urine testing chip successfully proven to measure the urine’s water content using a CSRR coupled with a microstrip and a microfluidic channel. The CSRR is a resonant kind of electromagnetic detector with complicated material permittivity sensitivity. Additionally, when the content of the urine changes, the complex permittivity of the urine sample under test changes. Results indicate that when the urine color darkened from the loss of water, the resonant frequency increased. As a result, the CSRR’s microwave approach is appropriate for identifying urine and tracking hydration levels.

In [27], researchers designed, fabricated, and tested a microstrip extremely sensitive differential sensor for the complicated permittivity characterization of urine samples. The operating frequency of the unloaded sensor is 1.25 GHz, and the sensing region has two pairs of open stub resonators. The sensor may be simply installed on a substrate made of inexpensive FR-4 Epoxy that is 1.6 mm thick. The readings are unaffected by the mounting of a Teflon beaker on the sensor. To quantify the frequency fluctuation, liquid mixes of water and urine at various concentrations were added to the suggested sensor. With a step size of 3.226%, the proportion of water in the combination ranged from 0% (100% pee) to 100% (0% urine), creating 32 data groups for the simulated findings. The following mixes were tested for validity: The experimentation involved varying concentrations of urine and water, including 0% urine (100% water), 20% urine (80% water), 33% urine (66% water), 50% urine (50% water), 66% urine (33% water), and 100% urine (0% water). To attain a sensing sensitivity of approximately 3%, the complex permittivity of the samples under investigation was determined through nonlinear least square curve fitting conducted in MATLAB 2017a.

The research in [28] suggested a biosensor to monitor the amount of glucose, albumin, and urea in blood and urine as well as to identify blood components. The simple adjustment of this sensor, which was also illustrated in the current essay, is one of its most crucial characteristics. We configured the sensor to function at low frequencies (about 1 THz) and high frequencies (approximately 193 THz), and then we tested several samples to demonstrate this crucial characteristic. According to the modeling results, the biosensor exhibits its maximum sensitivity at 136 μm/RIU at low frequency and 500 nm/RIU at high frequency. Additionally, the figure of merit (FoM) stands at 2000 for high frequency and 155 for low frequency. Noteworthy attributes of this biosensor include real-time measurement capability, high speed, compact size, cost-effectiveness, and the ability to label samples without any additional charge.

In [29], created research to see whether urine color (UCOL), a simple and affordable approach, was a reliable way to determine urine concentration. All women, including pregnant, lactating, and control women, showed a statistically significant correlation between 24-h UCOL and 24-h UOSM (r=0.61−0.84, all *p* 0.001). Using a statistical study of the receiver operating characteristic, the study reveals that the Urea Creatinine Osmolality ratio (UCOL) corresponding to this threshold was marked at 4 on the UCOL chart. Furthermore, it was found that both the 24-h and single-sample UCOL measurements demonstrated high diagnostic accuracy in identifying UOSM (urine osmolality) of 500 mOsm/kg in all women, with the area under the curve ranging from 0.68 to 0.95 (*p* values ranging from 0.001 to 0.46).

The researchers in [30] developed a sandwich format (uric acid) UA biosensor that produced accurate findings when used with chronoamperometry for urine analysis. To the best of our knowledge, this is the most straightforward UA biosensor that has been documented. It operates in the chronoamperometric mode, inhibits direct uric acid oxidation, and addresses issues with possible urine interferences. It should be noted that these biosensors were made in-house utilizing screen-printing technology, which enables inexpensive mass manufacturing, especially because water-based carbon ink was utilized.

In [31], the validity and effectiveness of two innovative techniques—thirst feeling and urine volume—were examined in this study to determine the level of hydration in 29 active males (mean SD; age, 23; 4 years body mass, 76.02; 11.94 kg) when they were at rest. There were eight different combinations of the four different water challenges (4.8,9.3,11.0, or 14 mL/kg) and the two different hydration states (mildly Hypohydrated (HY), −2.0%, and Euhydrated (EU), −0.2% body mass). The research concludes that healthy men may use straightforward assessments of morning thirst and urine volume to detect moderate dehydration and direct fluid replenishment. These two methods are important since the approximate threshold for the start of thirst, decreased endurance exercise performance, and declines in working memory and mood is HY (−2% body mass).

According to [32], the permittivity of urine will be used in this investigation to see whether there are any significant differences between the various stages of breast cancer. Over a frequency range of 10 MHz to 20 GHz, variations in the electrical resistance of urine samples to applied microwaves were observed. This is done by utilizing a network vector analyzer to measure how samples react to applied microwave radiation. The statistically significant variation in urine permittivity levels across various stages of breast cancer was discovered using SPSS. The findings reveal a considerable variation in all-dielectric parameters of urine permittivity between stage 1 and stage 2.

## 3. Proposed Framework

**Figure 1:**
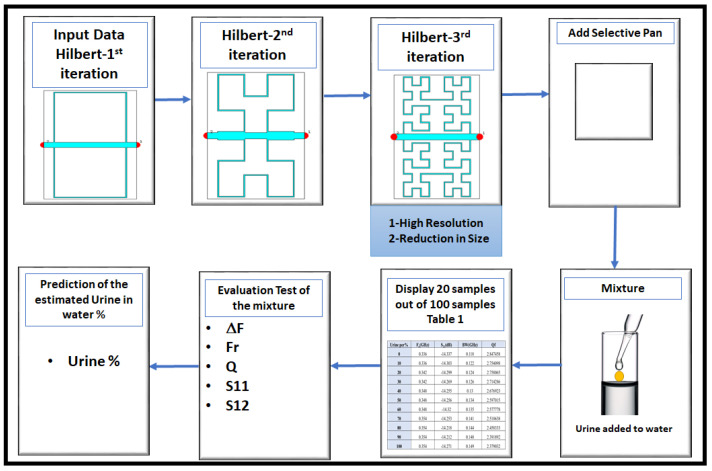
Proposed Scheme Based on Hilbert Cell.

The general proposed framework, as shown in Figure 1, has seven stages. The first three stages represent the first, second, and third iterations of the Hilbert cell. In the fourth stage, we added the selective pan, which is created to include the mixture of urine drops that are added to the water, which represents the fifth stage. In addition, a table contains twenty samples out of one hundred samples, which is explained in Table 1. Then, numerical analysis is employed for the evaluation of the mixture in stage six, Finally, stage seven produces the prediction of the estimated urine in the water in percentage.

## 4. Technical Specifications of the Sensor’s Shape and Structure

**Figure 2:**
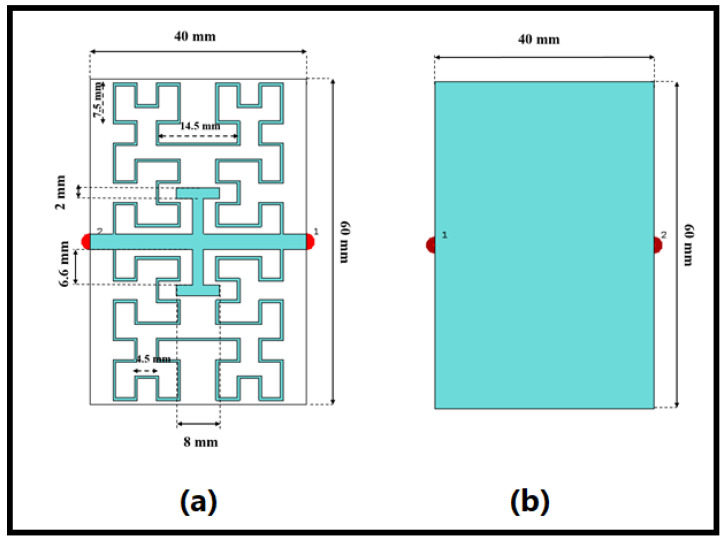
The front and back views of the proposed sensor, in (**a**) and (**b**), respectively.

The author-suggested sensor structure, based on Hilbert fractal geometry, is covered in this section. Three major components put on an Epoxy Glass FR4 substrate make up the sensor’s design. The design then includes Hilbert geometry third iteration inserts as high capacitive surfaces [33] to guarantee the field leakage from the SUT across a large area. The T-shaped resonator, which is the last component, is included to keep the suggested sensor’s frequency resonance in the lower frequency region of interest [34]. The copper resonator layer, which can be seen in the front view of the substrate, is 0.035 mm thick. To achieve great penetration through the skin depth criteria, the scientists built their sensor to operate at a frequency of 0.46 GHz [35]. On the other hand, including the T-shaped resonator with the Hilbert inclusions would result in a considerable improvement in the effective permittivity ($\varepsilon_{r}$). In order to further characterize the SUT’s dielectric characteristics, the intensity of the induced electric field [36] would increase. As shown in Figure 2b, the proposed sensor is supported by a complete ground plane built on a copper layer. The fractal Hilbert curve is shown in Figure 2. The transmission line is connected to a continuous line, which forms the fractal Hilbert curve. The fractal curve is fit in a rectangular section of S as external side.

By increasing the iteration level *k* of the curve, one reduces the elemental grid size as $\frac{S}{\left(2^{k}-1\right)S}$; the space between lines diminishes in the same proportion, and the length of the curve increases as expressed in Equation (1):

(1) $$L\left(k\right)=\left(2^{k}+1\right)S$$

To contain the resonator in the smallest practical area, it is ideal to increase the Hilbert curve’s iteration as much as possible. However, there is a trade-off between a Hilbert resonator’s quality factor (Qf) and miniaturization (curves with high *k*). The factors that define this tradeoff for a microstrip resonator are the width of the strip (*w*) and the gap (*g*) between the strips [37]. Both dimensions (*w* and *g*) are connected with the external side *S* and iteration level *k*(k⩾2) by using Equation (2):

(2) $$S=S^{k}\left(w+g\right)-g$$

It is obvious from this equation that attempting to achieve high levels of miniaturization implies lower values of *w*, resulting in an increase in dissipation losses and a decrease in the quality factor. Keep in mind that if *S* and the ratio w/g are maintained constant, *w* virtual halves from *k* to k+1 [37].

## 5. Hilbert Cell Characterizations

**Figure 3:**
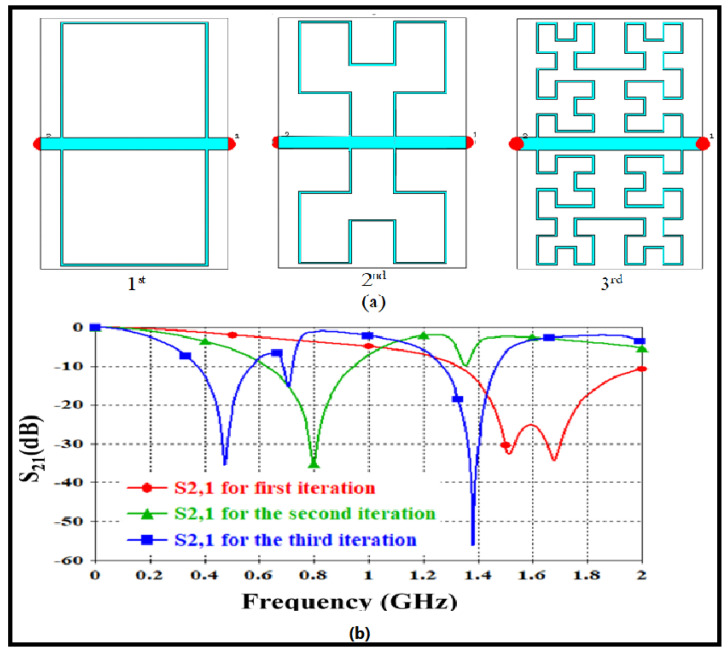
Exploration of parameters through altering Hilbert iterations: (**a**) Different Hilbert iterations surrounding the geometric models of the transmission line. (**b**) Changes in S21 spectra corresponding to operational variations.

In Figure 2a, the proposed Hilbert structure is depicted, constructed using a second iteration fractal geometry. The outer dimensions of the unit cell measure $32.0\times 27.8\times 0.035\;{\mathrm{mm}}^{3}$ which is the dimension describing the size of the resonator layer for the distance from the transmission line to the end of the resonator layer. To prevent cross-line intersections and radiation leakage from the trace width, a 0.5 mm width polygonal conductive trace is utilized, following recommendations by Mathew et al. (2013) [38]. To evaluate the performance of the proposed fractal when mounted on an FR4 substrate in terms of dispersion diagram and S-parameters, a 3D full-wave analysis is carried out using the CST MWS-v2 software package. The authors conducted their analysis parametrically in this study. Subsequently, the impact of varying the Hilbert iteration on the performance of the proposed unit cell is investigated. The configuration and arrangement of the unit cell surrounding the proposed transmission line center are illustrated in Figure 2a. S21 spectra are observed concerning the increase in fractal iteration, as depicted in Figure 3a. From the S21 spectra in Figure 3b, it is evident that the first iteration for the proposed geometry exhibited a frequency band around 1.6 GHz with a high band and low quality factor. However, increasing the Hilbert order to the second iteration resulted in two frequency bands around 0.8 GHz, with S21 approximately −36 dB, and 1.4 GHz, with S21 approximately −10 dB. The third iteration revealed three modes at 0.43 GHz, with S21 approximately −37 dB, 0.66 GHz, with S21 approximately 15 dB, and 1.28 GHz, with S21 approximately −58 dB, as observed in Figure 3b.

## 6. Design of the Container

**Figure 4:**
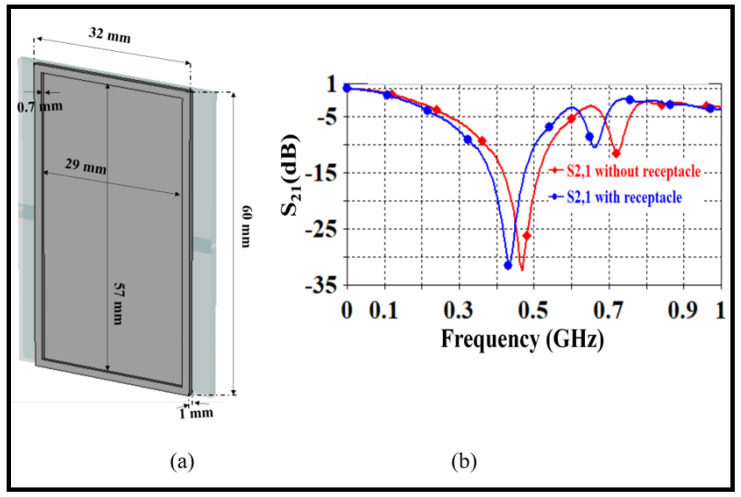
(**a**) Geometrical details of receptacle (**b**) S21 spectra.

The receptacle needs to encompass the entire Hilbert structure area to facilitate field penetration to the Sample Under Test (SUT). As depicted in Figure 4a, a rectangular shape measuring $60\times 32\times 1\;{\mathrm{mm}}^{3}$ is suggested for the receptacle. This choice of geometry is based on observed electric field intensity distributions, which will be discussed later. Simulation results obtained using CST MWS revealed a resonant frequency shift of approximately 20 MHz, as illustrated in Figure 4b.

## 7. Approach to Design and Thorough Investigation

In this section, we outline the design development process, aiming to illustrate the steps taken to achieve optimal performance for the operation.

### 7.1. Development of the Transmission Line

**Figure 5:**
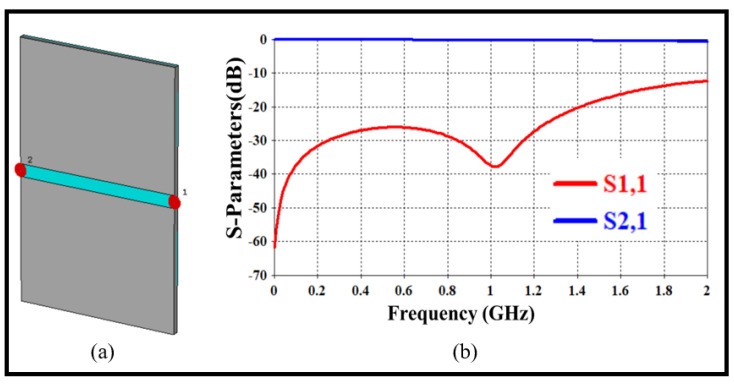
(**a**) Transmission line structurer, (**b**) S-parameters spectra for the transmission line.

We crafted a 50 Ω transmission line, as depicted in Figure 5a, to guarantee smooth power transfer from port 1 to port 2. Subsequently, S21 spectra are analyzed using CST MWS, as shown in Figure 5b.

**Figure 6:**
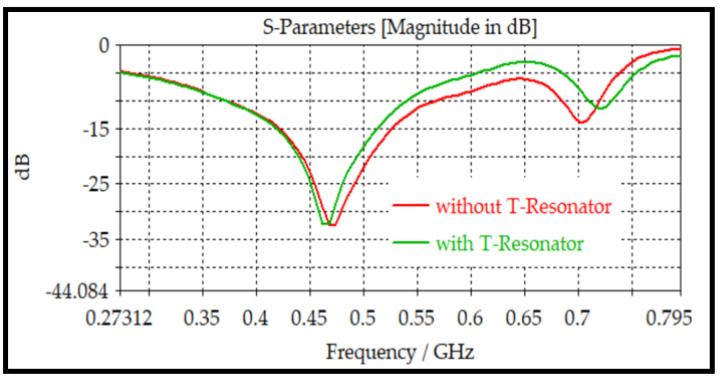
The frequency characteristics of the selected sensor configuration with and without T-resonator.

A t-resonator is included in the design to increase the sensor sensitivity by increasing the interacting area to the material under test, whereas the T-Resonator has little effect on the resonant frequency. Figure 6 shows the effect of the T-Resonator on the resonant frequency.

### 7.2. Electric Field Distribution with Different Depths of the Container

**Figure 7:**
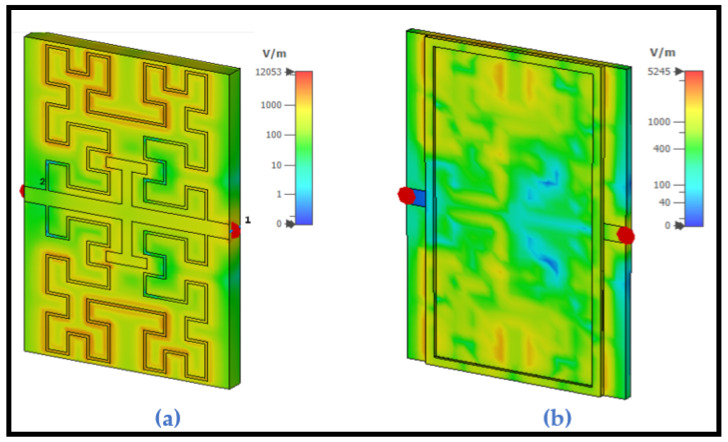
Electric field simulation from port 1 (**a**) without the pan at the resonant frequency 0.46 GHz and (**b**) with the pan at the resonant frequency 0.432 GHz.

Figure 7 shows electric field simulation from port 1 (a) without the pan at the resonant frequency 0.46 GHz and (b) with the pan at the resonant frequency 0.432 GHz. The heightened depth with the base of the container significantly alters both the resonant frequency and field distribution, consequently exerting a substantial influence on the sensing process.

**Figure 8:**
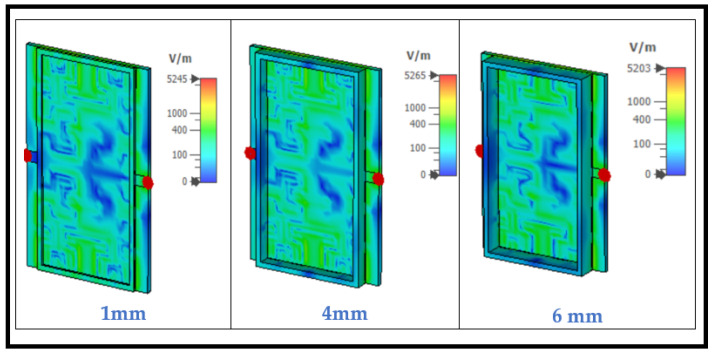
Electric field distribution at the resonant frequency for different depths of the container.

Increasing the depth while maintaining a constant base has a negligible effect on the distribution of the electric field seen in Figure 8, which is a crucial advantage, as it allows the proposed sensor to operate with extremely minimal sample sizes of a mixture of urine and water for detection.

**Figure 9:**
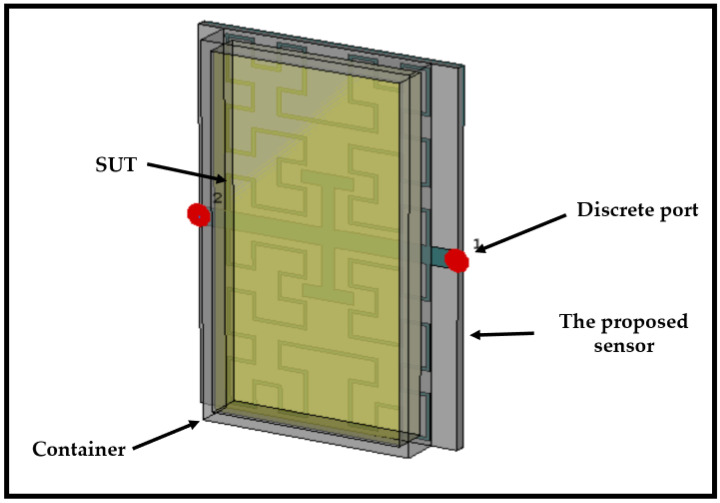
Sensor with material under test.

Inaddition, Figure 9 shows sensor with material under test.

## 8. Quantitative Evaluation

This study assesses one hundred data points, incrementally adding one percent of urine to water in each point, up to a hundred percent, with a step size of one percent. The proportion of water diminishes in tandem with the increase in urine concentration. The findings presented in Table 1 highlight the impact of escalating urine concentration on the resonance frequency, surpassing the reference value. Moreover, this escalation influences both the sensor’s performance and the quality factor. Manipulating the resonance frequency directly impacts the quality factor (Qf), emphasizing its sensitivity to urine concentration variations.

We have noticed that as the urine percentages increase, the resonant frequency tends to shift primarily towards lower frequency bands. This occurs because the effective permittivity value of the mixture increases rapidly with higher urine percentages, which is attributed to changes in the dielectric constant. The constant of the urine–water mixture is determined using the relationship provided in the study by Krzysztofik et al. (2013) [39].

**Figure 10:**
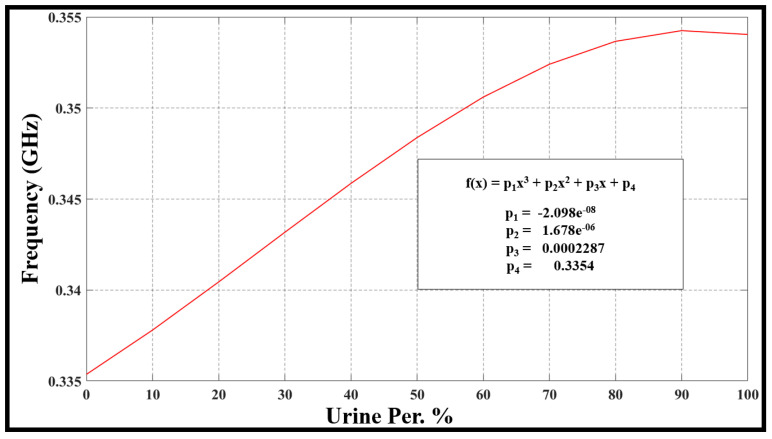
The model describes changes in the resonance frequency concerning urine content variation in percentage.

**Figure 11:**
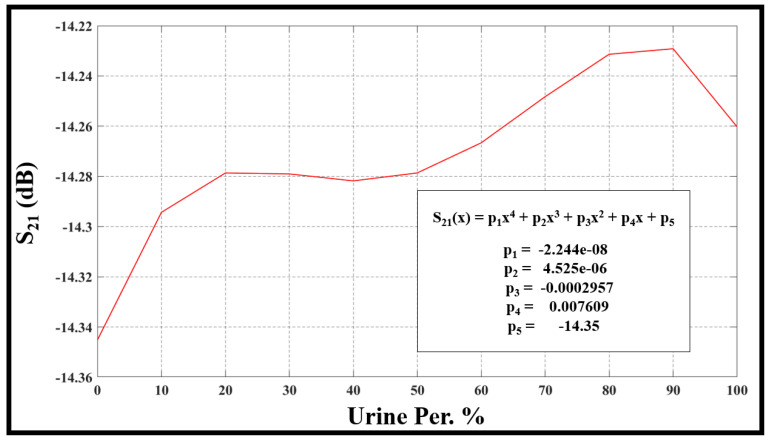
The model describes changes in the $S_{21}$ in terms of urine content variation in percentage.

**Figure 12:**
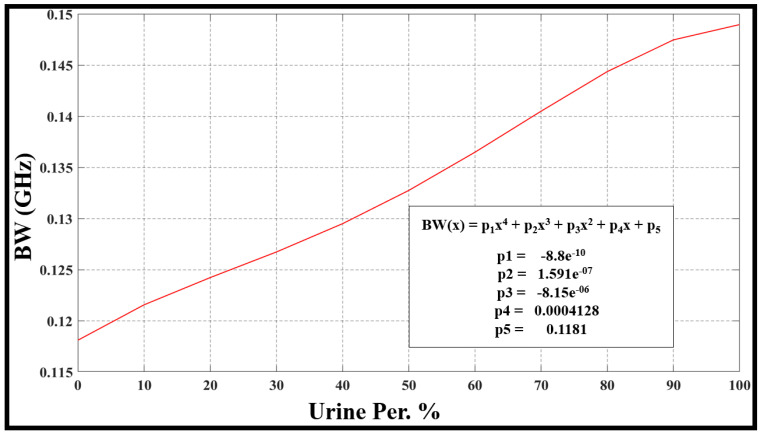
The model illustrates how the bandwidth changes with variations in the percentage of urine content.

**Figure 13:**
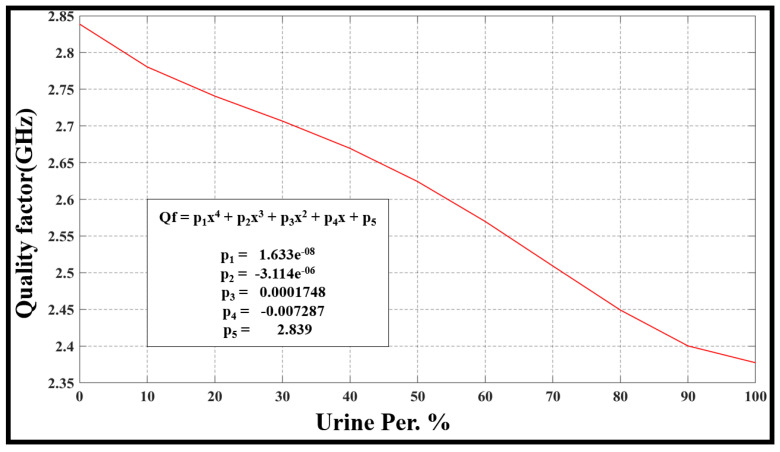
The model describes changes in the Quality Factor (QF) concerning urine content variation in percentage.

The evaluation of frequency resonance change, bandwidth, sensor performance, and Q-factor relies on the fluctuations in urine percentage within water. This is directly linked to alterations in the concentration of the urine–water mixture. The Figure 10, Figure 11, Figure 12 and Figure 13 refer to the simulation results (fo, S21, BW, and Qf) with different urine percentages. Figure 10 presents a polynomial relationship derived from a curve fitting model to describe the resonance frequency change in relation to urine content within the SUT.

(3) $$\varepsilon_{mixt}^{a}=\sum_{i}^{\;}f_{i}\;\varepsilon_{i}^{a}=\varepsilon_{r}=\varepsilon \left(r_{water}\right)\times \mathrm{percentage}\;\mathrm{of}\;\mathrm{water}+\varepsilon \left(r_{urine}\right)\times \mathrm{percentage}\;\mathrm{of}\;\mathrm{urine}$$

The factors $p_{0}$, $p_{1}$, $p_{2}$, and $p_{3}$, provided in the equation within Figure 10, represent unknown coefficients that establish a comprehensive relationship between resonance frequency and urine content in samples, once accurately determined. The equation can be solved through curve fitting in MATLAB, enabling the identification of unknown variables, as detailed in Figure 10, which shows the model describing changes in the resonance frequency in terms of urine content variation in percentage. This relationship can be estimated as a polynomial equation, which is defined inside Figure 10 with their parameters.

In addition, Figure 11 illustrates the model describing changes in the sensor in terms of urine content variation in percentage. A polynomial equation defining this relationship and its parameters can be found inside Figure 11.

Furthermore, Figure 12 depicts the model describing changes in the bandwidth (BW) in terms of urine content variation in percentage. Figure 12 has a polynomial equation that describes this connection and its parameters.

Moreover, Figure 13 shows the model describes changes in the Quality Factor (QF) in terms of urine content variation in percentage. Inside Figure 13, the reader will discover a polynomial equation that explains this relationship and its parameters.

**Figure 14:**
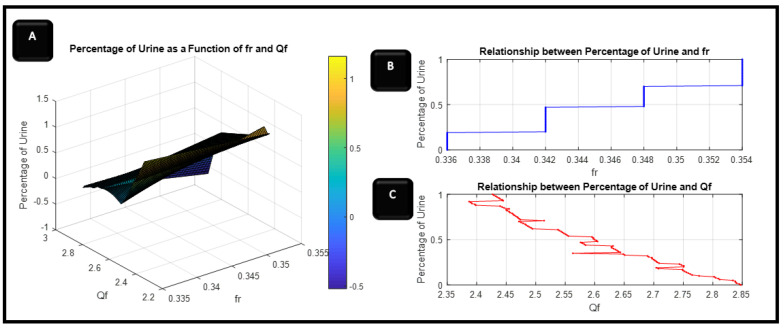
(**A**) the relationship between the percentage of urine as a function of the quality factor and resonance frequency, (**B**,**C**) from show the relationship separately between the percentage of urine with fr and with Qf.

Furthermore, Figure 14 illustrates the relationship between the percentage of urine as a function of the quality factor and resonance frequency, as explained in Part A of Figure 14. Both Parts B and C from Figure 14 show the relationship separately between the percentage of urine and fr alone and Qf. Figure 14B presents the relation between the resonant frequency and different urine percentages from (1–100) % in water: for the first ten samples, the resonant frequency does not change, then it changes by 6 MHz for the next ten percentages. For the urine samples from 40 to 60, the resonant frequency remain constant, then the resonant frequency increase by 6 MHz for the next. Finally, the resonant frequency increased by 6 MHz for percentages from 60 to 70% to remain constant for percentages from 70 to 100%.

Table 2 shows the measurements of various combination percentages in practice with the suggested sensor. This table presents the results of practical measurements conducted using a newly proposed sensor technology across various mixture percentages. The sensor is tested in real-world conditions to assess its effectiveness in accurately detecting and measuring different mixtures. The data displayed in the table showcases the sensor’s performance across a range of mixture compositions, providing valuable insights into its potential applications and reliability in diverse scenarios.

Table 3 shows the comparisons between the proposed work with other state-of-the-art methods. This comparative table illustrates the principle of the sensor design and the frequency range for various types of liquid samples. In addition, this comparison is essentially in terms of the reference used, resonators type, resonant frequency (GHz), SUT, Area (${\mathrm{mm}}^{2}$) and s-parameters, respectively. According to the outcomes of Table 3, the proposed work demonstrates that it outperforms other related work, as highlighted in yellow in the red bold text.

## 9. Conclusions

This research introduced a microwave sensor designed for liquid analysis, operating as a compact resonator with Hilbert’s fractal structure. A T-Resonator was included in the design to increase the interacting area with the SUT, and it also had a negligible effect on the resonant frequency. Printed on an FR4 substrate, the sensor measured $40\times 60\times 1.6\;{\mathrm{mm}}^{3}$. The container was made from the same material as the substrate. To assess its capabilities, the sensor underwent testing with urine samples, evaluating its performance across 100 samples with different urine concentrations. The sensor was able to detect the urine level accurately, even though the sample was small. We did not need to increase the depth of the container to accommodate more quantity.

In future work, there is a plan to use the proposed sensor on a flexible substrate for real-time testing due to the fast-growing demands of the industry. Therefore, we are planning to test the sensor with different organic and non-organic fluids, applying the obtained measurements to be expressed in the application of biological fluids using the same proposed technology. We intend to implement the sensor into practical use and then conduct practical tests to compare our results with the theoretical findings.

## Figures and Tables

**Table 1:** Urine Effect of the Percentage of the Resonance Frequency, Transmitted Coefficient S21, and Quality Factor.

Urine per%	Fo (GHz)	S21 (dB)	BW (GHz)	Qf
0	0.336	−14.337	0.118	2.847458
10	0.336	−14.303	0.122	2.754098
20	0.342	−14.299	0.124	2.758065
30	0.342	−14.269	0.126	2.714286
40	0.348	−14.255	0.13	2.676923
50	0.348	−14.256	0.134	2.597015
60	0.348	−14.32	0.135	2.577778
70	0.354	−14.253	0.141	2.510638
80	0.354	−14.218	0.144	2.458333
90	0.354	−14.212	0.148	2.391892
100	0.354	−14.271	0.149	2.379032

**Table 2:** Practical measurements of different mixture percentages using the proposed sensor, where the first row in the table represents No. of water samples (W%), density of water (DoW) is measured in (kg/m^3^), thermal conductivity of water (THCoW) is measured in (W/K/m), electric conductivity of water (ECoW) is measured in, density of urine (DoU) is measured in (kg/m^3^), thermal conductivity of urine (THCoU) is measured in (W/K/m), electric conductivity of urine (ECoU) is measured in (S/m), percentage of urine (U%), R represents $\varepsilon_{r}$ for mixture urine with water, DR refers to the density of mixture, and THR refers to the thermal conductivity of the mixture.

SNo.	Water	DoW	THCoW	ECoW	W%	U	DoU	THCoU	ECoU	U%	R	DR	THR	ER
1	78	1000	0.6	1.59	1	50	1024	0.56	1.75	0	78	1000	0.6	1.59
2	78	1000	0.6	1.59	0.9	50	1024	0.56	1.75	0.1	75.2	1002.4	0.596	1.606
3	78	1000	0.6	1.59	0.8	50	1024	0.56	1.75	0.2	72.4	1004.8	0.592	1.622
4	78	1000	0.6	1.59	0.7	50	1024	0.56	1.75	0.3	69.6	1007.2	0.588	1.638
5	78	1000	0.6	1.59	0.6	50	1024	0.56	1.75	0.4	66.8	1009.6	0.584	1.654
6	78	1000	0.6	1.59	0.5	50	1024	0.56	1.75	0.5	64	1012	0.58	1.67
7	78	1000	0.6	1.59	0.4	50	1024	0.56	1.75	0.6	61.2	1014.4	0.576	1.686
8	78	1000	0.6	1.59	0.3	50	1024	0.56	1.75	0.7	58.4	1016.8	0.572	1.702
9	78	1000	0.6	1.59	0.2	50	1024	0.56	1.75	0.8	55.6	1019.2	0.568	1.718
10	78	1000	0.6	1.59	0.1	50	1024	0.56	1.75	0.9	52.8	1021.6	0.564	1.734
11	78	1000	0.6	1.59	0	50	1024	0.56	1.75	1	50	1024	0.56	1.75

**Table 3:** Comparisons of the proposed work with other related work.

Ref	Resonators Type	Resonant freq./GHz	SUT	Area/mm^2^	s-Parameters
[2]	1st order of Minkowski open loops	2.45	blood glucose level	25 × 50	−23
[4]	Two-wire line (in UWSNP-1M) or a coaxial line (in UWSNP-2M) short-circuited at the opposite end	1.06	water content in flowing crude oil	-	-
[10]	interdigital capacitor	1.35	different types of water	60 × 40	−18
[11]	microstrip transmission line	2 to 3	moisture content in oil	50 × 100	-
[15]	complementary split-ring resonator (CSRR)	2.461	Dielectric (Rogers RO 6006 and FR4) and magneto-dielectric (composite materials based on rubber and carbonyl-iron with 30 and 45 weight percentages of carbonyl-iron)	-	−26
[16]	Complementary Circular Spiral Resonator (CCSR)	2.4	mixture of ethanol and water	25 × 30	−15
[19]	complementary split ring resonator (CSRR)	2.54	Oil Samples	-	−24.4
[24]	multiple complementary split-ring resonator (MCSRR)	2.45	mixture of ethanol and water	35 × 25	−23
[25]	CSRR	3.99 to 4	Urine	38 × 26	−16
[27]	optical sensors	2	Blood and Urine Components	50 × 50	-
[34]	Two-element antenna with fractal isolator	8 to 30	-	23 × 23	-
Proposed work	Hilbert structure	0.46	Urine	60 × 40	−34

## Data Availability

The data presented in this study are available on request from the corresponding author.
